# The UNITE database for molecular identification and taxonomic communication of fungi and other eukaryotes: sequences, taxa and classifications reconsidered

**DOI:** 10.1093/nar/gkad1039

**Published:** 2023-11-11

**Authors:** Kessy Abarenkov, R Henrik Nilsson, Karl-Henrik Larsson, Andy F S Taylor, Tom W May, Tobias Guldberg Frøslev, Julia Pawlowska, Björn Lindahl, Kadri Põldmaa, Camille Truong, Duong Vu, Tsuyoshi Hosoya, Tuula Niskanen, Timo Piirmann, Filipp Ivanov, Allan Zirk, Marko Peterson, Tanya E Cheeke, Yui Ishigami, Arnold Tobias Jansson, Thomas Stjernegaard Jeppesen, Erik Kristiansson, Vladimir Mikryukov, Joseph T Miller, Ryoko Oono, Francisco J Ossandon, Joana Paupério, Irja Saar, Dmitry Schigel, Ave Suija, Leho Tedersoo, Urmas Kõljalg

**Affiliations:** Natural History Museum, University of Tartu, Vanemuise 46, 51003 Tartu, Estonia; Department of Biological and Environmental Sciences, University of Gothenburg, Box 453, 405 30 Göteborg, Sweden; Gothenburg Global Biodiversity Centre, University of Gothenburg, Box 453, 405 30 Göteborg, Sweden; Gothenburg Global Biodiversity Centre, University of Gothenburg, Box 453, 405 30 Göteborg, Sweden; Natural History Museum, University of Oslo, Box 1172 Blindern, 0318 Oslo, Norway; The James Hutton Institute, Craigiebuckler, Aberdeen AB15 8QH, UK; Institute of Biological and Environmental Sciences, University of Aberdeen, Cruickshank Building, St Machar Drive, Aberdeen AB24 3UU, UK; Royal Botanic Gardens Victoria, Birdwood Avenue, Melbourne, VIC 3004, Australia; Global Biodiversity Information Facility (GBIF), Secretariat, Universitetsparken 15, DK-2100 Copenhagen Ø, Denmark; Institute of Evolutionary Biology, Faculty of Biology, University of Warsaw, ul. Zwirki i Wigury 101, 02-089 Warsaw, Poland; Swedish University of Agricultural Sciences, Department of Soil and Environment, Box 7014, SE-750 07 Uppsala, Sweden; Natural History Museum, University of Tartu, Vanemuise 46, 51003 Tartu, Estonia; Institute of Ecology and Earth Sciences, University of Tartu, J. Liivi 2, 50409 Tartu, Estonia; Royal Botanic Gardens Victoria, Birdwood Avenue, Melbourne, VIC 3004, Australia; Westerdijk Fungal Biodiversity Institute, The Netherlands; National Museum of Nature and Science, Japan; Botany Unit, Finnish Museum of Natural History, P.O.Box 7, 00014 University of Helsinki, Finland; Natural History Museum, University of Tartu, Vanemuise 46, 51003 Tartu, Estonia; Natural History Museum, University of Tartu, Vanemuise 46, 51003 Tartu, Estonia; Natural History Museum, University of Tartu, Vanemuise 46, 51003 Tartu, Estonia; Institute of Ecology and Earth Sciences, University of Tartu, J. Liivi 2, 50409 Tartu, Estonia; School of Biological Sciences, Washington State University, 2710 Crimson Way, Richland, WA 9935, USA; Institute of Ecology and Earth Sciences, University of Tartu, J. Liivi 2, 50409 Tartu, Estonia; Department of Biological and Environmental Sciences, University of Gothenburg, Box 453, 405 30 Göteborg, Sweden; Global Biodiversity Information Facility (GBIF), Secretariat, Universitetsparken 15, DK-2100 Copenhagen Ø, Denmark; Department of Mathematical Sciences, Chalmers University of Technology, Gothenburg, Sweden; Institute of Ecology and Earth Sciences, University of Tartu, J. Liivi 2, 50409 Tartu, Estonia; Global Biodiversity Information Facility (GBIF), Secretariat, Universitetsparken 15, DK-2100 Copenhagen Ø, Denmark; Department of Ecology, Evolution, and Marine Biology, University of California at Santa Barbara, USA; Biome Makers Inc., Davis, CA, USA; European Molecular Biology Laboratory, European Bioinformatics Institute, Hinxton, Cambridge, UK; Natural History Museum, University of Tartu, Vanemuise 46, 51003 Tartu, Estonia; Institute of Ecology and Earth Sciences, University of Tartu, J. Liivi 2, 50409 Tartu, Estonia; Global Biodiversity Information Facility (GBIF), Secretariat, Universitetsparken 15, DK-2100 Copenhagen Ø, Denmark; Natural History Museum, University of Tartu, Vanemuise 46, 51003 Tartu, Estonia; Institute of Ecology and Earth Sciences, University of Tartu, J. Liivi 2, 50409 Tartu, Estonia; Institute of Ecology and Earth Sciences, University of Tartu, J. Liivi 2, 50409 Tartu, Estonia

## Abstract

UNITE (https://unite.ut.ee) is a web-based database and sequence management environment for molecular identification of eukaryotes. It targets the nuclear ribosomal internal transcribed spacer (ITS) region and offers nearly 10 million such sequences for reference. These are clustered into ∼2.4M species hypotheses (SHs), each assigned a unique digital object identifier (DOI) to promote unambiguous referencing across studies. UNITE users have contributed over 600 000 third-party sequence annotations, which are shared with a range of databases and other community resources. Recent improvements facilitate the detection of cross-kingdom biological associations and the integration of undescribed groups of organisms into everyday biological pursuits. Serving as a digital twin for eukaryotic biodiversity and communities worldwide, the latest release of UNITE offers improved avenues for biodiversity discovery, precise taxonomic communication and integration of biological knowledge across platforms.

## Graphical Abstract

**Graphical Abstract:**
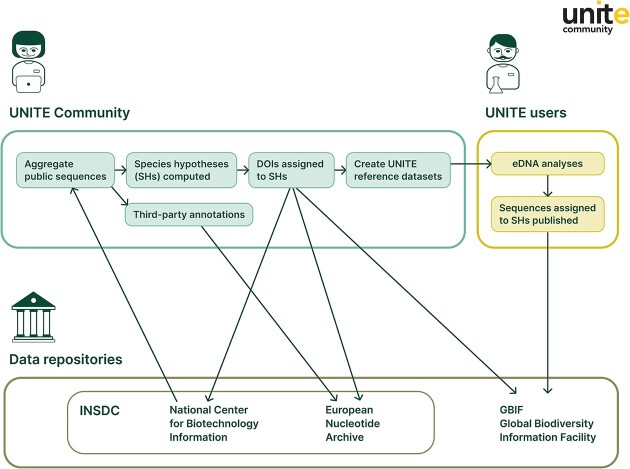

## Introduction

Knowledge on species identity is a cornerstone of biology and provides key information for understanding biodiversity changes driven by climate change and other human pressures. Such taxonomic knowledge has traditionally been obtained primarily from sources such as field surveys by skilled practitioners with substantial experience in morphological studies and taxonomy, but the last few decades have seen a steady increase in the use of molecular (DNA sequence) tools for characterization of biodiversity. DNA sequences from substrates such as soil and water invariably indicate a significantly larger extant biodiversity than known from traditional approaches. Indeed, many of the species and evolutionary lineages recovered in this way are, so far, only known from sequence data. Molecular surveys thus bring many pressing questions to the fore, notably how to root environmental DNA sequences at the species level if there is no other descriptive information, and how to communicate species that may lack formal names and taxonomic affiliations all the way up to the kingdom level. Furthermore, many of these studies suggest novel, poorly understood biological associations and co-occurrences among organisms across distinct groups, questioning the current practice of routinely singling out particular groups – such as fungi – for environmental sequencing.

The UNITE database (https://unite.ut.ee) was launched in 2003 as a Sanger sequence-oriented online resource for molecular identification of fungi. It is focused on the ∼600-base nuclear ribosomal internal transcribed spacer (ITS) region, the formal fungal DNA barcode (1), and includes all public ITS sequences from the International Nucleotide Sequence Databases Collaboration (INSDC; (2)) plus ITS sequences supplied from UNITE users and partners. The sheer number of unidentified, and for all practical purposes unidentifiable, fungal species recovered from environmental sequencing stimulated UNITE to devise the so-called species hypothesis (SH) concept. SHs represent an open and reproducible approach to unambiguously infer, identify and communicate described as well as undescribed species (3). UNITE defines SHs from public ITS sequences through a series of quality filtering and single-linkage clustering steps at successively more stringent threshold levels. All SHs, supplemented with their source metadata and trait information, are assigned a digital object identifier (DOI) to facilitate unambiguous scientific communication and ensure data interoperability across datasets and studies (Supplementary Figure S1). Over time, UNITE, together with its data management platform, PlutoF, has evolved along with DNA sequencing technologies into a fully-fledged online workbench and sequence management environment for handling not only sequence identification but most steps in DNA barcoding and metabarcoding studies. UNITE offers web-based third-party sequence curation and addition of metadata, and SH-based reference datasets are released for many popular metabarcoding (massive parallel sequencing of amplified genetic markers; (4)) pipelines, notably QIIME (5) and SINTAX (6).

The rapid development of high-throughput sequencing methods and the scope of the biological questions that are being addressed in its wake provoke a reconsideration of many aspects of biological research. While assignment of a DOI to an otherwise nameless species ensures scientific reproducibility of that species and its metadata, it does little to address or clarify the higher-level classification of that species. As a result, metabarcoding and taxonomy are often pursued as two essentially distinct disciplines where progress in one is not being incorporated into the other. Furthermore, the fact that many of these nameless species cannot be grown away from their natural habitat hints at currently unknown biological associations, putatively across organism groups and points to a limitation of the current routine use of single-taxon metabarcoding efforts and databases (7). In parallel, large international biodiversity informatics efforts converge on systems for information dissemination and data exchange about our living world—systems to which individual metabarcoding efforts typically do not contribute at present. In this study we report on recent UNITE developments to refine the discovery potential and maximize the scientific usefulness of metabarcoding data against the backdrop of the massive increase in the volume and read length of environmental sequencing data.

## Databases

### Sequence data and quality control

UNITE synchronizes with the INSDC to download and update reasonably full-length Sanger-derived eukaryotic ribosomal DNA sequences on a quarterly basis. Additionally, it accepts user-provided Sanger-derived sequences and high-quality metabarcoding sequences, as long as certain criteria, such as minimum required length and detection of ribosomal gene regions, are met. At present, UNITE features >2.4M Sanger-derived and >7M metabarcoding sequences, the latter being representative sequences from operational taxonomic units (8), originating from the five large metabarcoding datasets (9–13) so far incorporated into UNITE. All sequences are subjected to a range of quality control steps, including the software tools ITSx (14) and UCHIME (15) to eliminate non-ITS and chimeric sequences, respectively. Other aspects of quality control are performed in a semi-automatic or manual way. For instance, sequences with clearly incorrect taxonomic annotations may be renamed automatically, whereas more subtle cases are flagged for manual examination. These various manual steps are very time-consuming, and artificial intelligence-based tools are currently explored to speed up these processes.

Other types of quality issues are presently not amenable to algorithmic interpretation. For instance, a sequence may be tagged with the wrong country of origin, or the name of a host may be misspelled. To facilitate the correction of such errors, UNITE offers web-based third-party sequence curation through the PlutoF biological data management environment (16). To date, >600 000 third-party annotations have been contributed by UNITE users, including >170 000 taxonomic re-annotations, >107 000 specifications of collection locality and >55 000 specifications of host and interacting taxa. Nearly 25 000 sequences have been identified as derived from nomenclatural types, and special weight is given to these sequences in subsequent sequence identification steps. Conversely, during the manual curation process, >13 000 sequences have been flagged for exclusion from active use due to unsatisfactory technical quality. Intragenomic ITS variability, to the extent that distinct ITS copies end up in different SHs, may potentially add noise in the estimation of biodiversity (17). UNITE keeps track of these copies through (living) specimen-based searches, and cases of non-trivial ITS variability can be accounted for by manually designating a more inclusive clustering threshold on a case-by-case basis. More statistics on third-party sequence curation by the UNITE community can be found at https://unite.ut.ee/curation.php, and a list of type-derived as well as low-quality sequences can be downloaded through PlutoF.

### Species and taxon hypotheses

**Figure 1.**
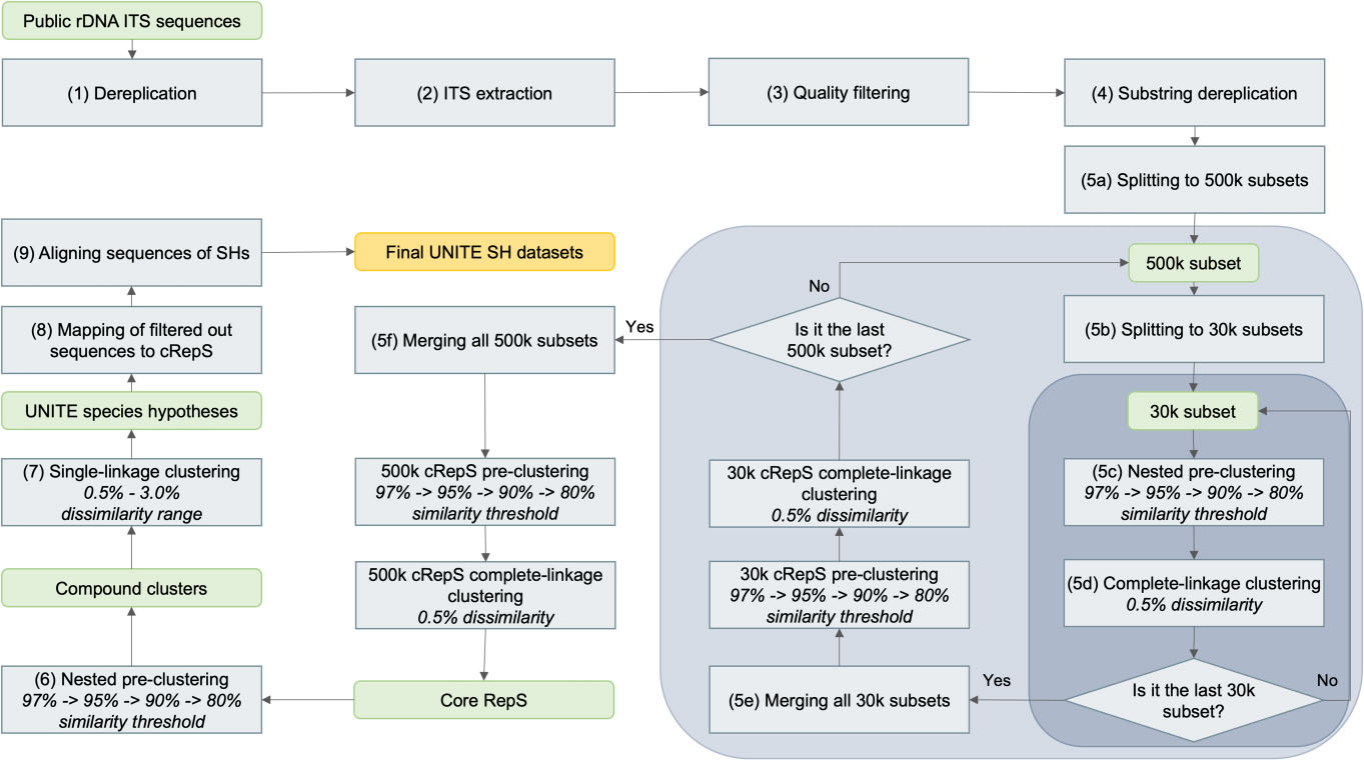
Diagram of the UNITE SH 9.0 calculation steps. The sequences are dereplicated using VSEARCH, and sequences that do not represent the full ITS region according to ITSx are dismissed. Following quality filtering, a series of successive clustering steps of generating subsets of 500 000 (500k) and 30 000 (30k) sequences and selecting core representative sequences (cRepS) is carried out. This yields what are termed ‘compound clusters’, which are sequence clusters roughly at the genus/subgenus level. These are further clustered into species hypotheses (SH). All clustering steps in the SH calculation workflow are performed using the USEARCH tool. The similarity thresholds (97%−95%−90%−80%) for the nested pre-clustering (5c, 6) were chosen to yield clusters at approximately the genus/subgenus level. A dissimilarity threshold (0.5%) for the complete-linkage clustering (5d) was selected to trim the dataset of closely related sequences around the core representative sequences. The core representative sequences undergo the final single-linkage clustering within a dissimilarity range of 0.5−3.0% with a 0.5% step. These dissimilarity thresholds were selected as the most commonly applied in species delimitation and sequence identification. For each SH, a representative sequence is selected, either automatically or based on prior manual curation. The species hypotheses are aligned to form the final SH datasets.

From its sequence data, UNITE infers species hypotheses (SHs) at six clustering dissimilarity thresholds (0.5, 1.0, 1.5, 2.0, 2.5 and 3.0% nucleotide divergence between SHs) to accommodate the dynamic nature of species boundaries across the target group. The ever-growing data volumes—primarily from metabarcoding data – prompted redesign and optimization of the SH inference process in various ways, notably using USEARCH (18) to sequentially cluster sequences into ever-smaller subsets, temporary dereplication of identical and near-identical sequences using VSEARCH (19) and the use of highly parallelized software tools in a high-performance computing environment (Figure 1).

UNITE taxon hypotheses (THs; (20)) are formed by mapping all SHs to the UNITE backbone classification through a taxon name selection algorithm that draws from all constituent sequences of each SH and tries to account for complications such as individual sequences with incompatible taxonomic annotations. Manually curated sequences are given extra weight in this process. Each TH is a dataset that contains all individuals and their ITS sequences from connected SHs. In addition, each dataset includes a distribution map, ecological traits and links to other associated THs. TH datasets are published with DataCite DOIs and are available as linkouts from SH DOIs. A visual example of a TH is shown as a screenshot in Supplementary Figure S2.

**Figure 2.**
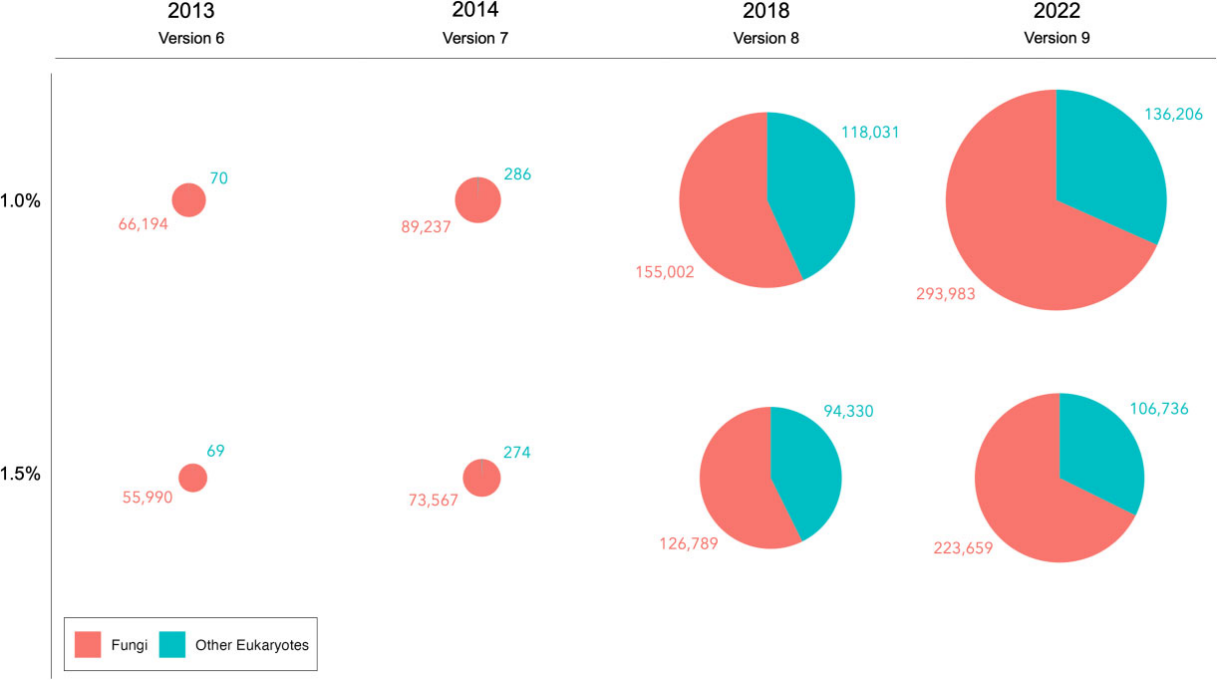
The number of species hypotheses at 1.0% and 1.5% between-species distance threshold through the four latest major versions of UNITE. Each SH is assigned a unique DOI every time the SHs are recomputed, and a versioning system keeps track of DOI names and contents over time, allowing users to follow how individual SHs are populated with sequences over time.

UNITE currently comprises 442 490 and 340 581 eukaryotic SHs at the 1.0% and 1.5% dissimilarity thresholds, respectively, and are based on 1 309 071 Sanger-derived sequences (of which 96% stem from the INSDC) and 6 825 264 metabarcoding sequences. The number of SHs grows rapidly over time (Figure 2). The share of metabarcoding sequences in the current UNITE release is 84%, and 47% and 45% (1.0% and 1.5% clustering dissimilarity, respectively) of all SHs are composed solely of metabarcoding sequences. Interestingly, 47% of all SHs consist of only Sanger-derived sequences, leaving a very modest 6–8% of the SHs composed of both metabarcoding and Sanger-derived sequences. Since all metabarcoding sequences in UNITE are representative sequences from non-singleton operational taxonomic units, no metabarcoding sequence in UNITE is a singleton in the strict sense of the concept (i.e. only one read in one sample). Even so, >2% of the SHs at the 1.5% threshold gap are formed by single metabarcoding (representative) sequences (7 568 SHs). The corresponding share of SHs composed of singleton Sanger-derived sequences is 31% (103  928 SHs). Sequences that are singletons for technical rather than biological reasons are likely to behave differently as clustering thresholds are relaxed, and we are looking into artificial intelligence-powered tools to further enhance the data quality over time.

### UNITE taxonomy

**Figure 3.**
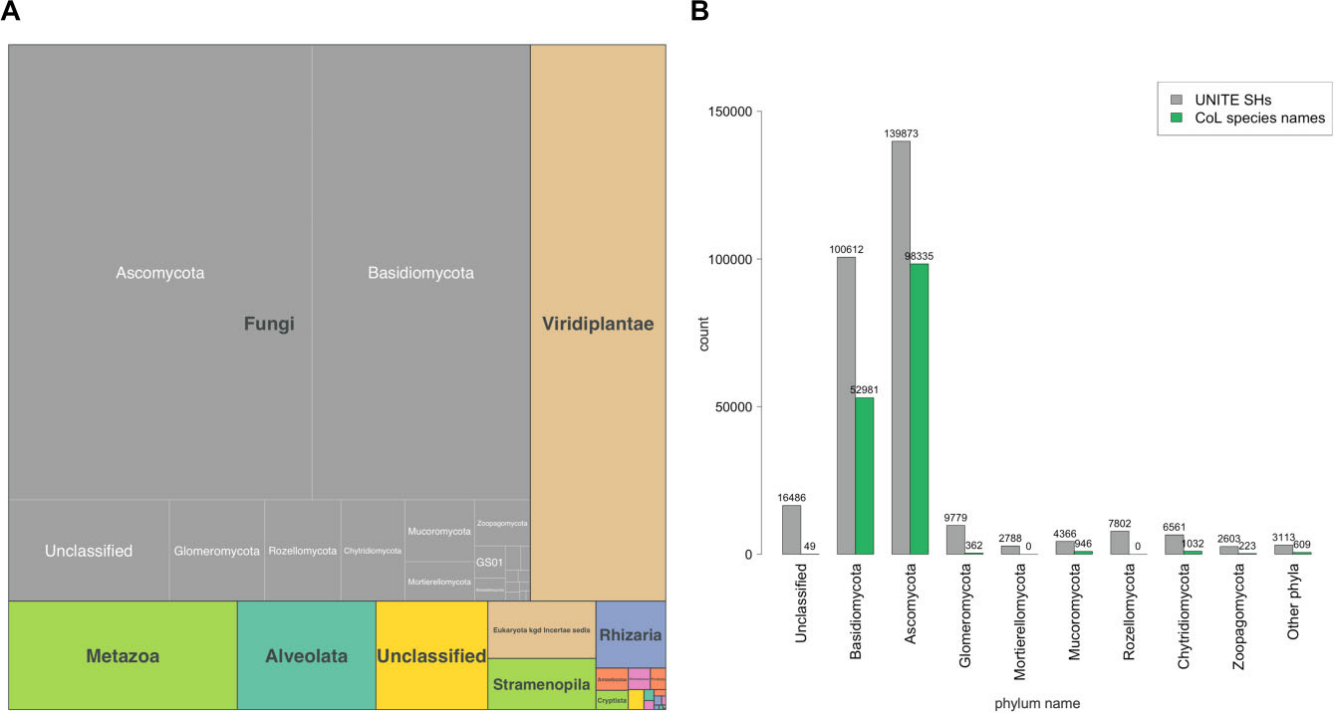
(**A**) Treemap of the most abundant taxa (kingdom and phylum) based on the taxonomy of UNITE SHs at 1.0% between-species distance threshold, (**B**) The number of UNITE SHs at 1.0% distance threshold versus species names per fungal phylum in the Catalogue of Life (CoL) checklist from 2023-06-29.

We have increased the taxonomic scope of UNITE from fungi to all eukaryotes, and UNITE now mirrors the INSDC for ‘Eukaryota’ rather than just ‘Eukaryota:Fungi’ (Figure 3). This makes UNITE useful for identifying more groups of organisms, for detecting and comparing the frequency of specific cross-kingdom associations in large datasets or sets of datasets and for highlighting non-target cross-kingdom PCR amplifications in single-group datasets (7). The pan-eukaryotic scope means that all eukaryotic SHs known from ITS sequence data—regardless of which classification level they are identified at—now have a persistent DOI to facilitate communication and metadata assembly across studies and datasets. The most well-represented kingdom is Fungi followed by Viridiplantae and Metazoa (Figure 3A). The number of fungal SHs exceeds the number of recognized fungal species names in Catalogue of Life (CoL; (21)) (Figure 3B), thus allowing the identification and communication of many undescribed species for which referencing across time and projects would otherwise be highly challenging. We hope to see a similar trend for other groups of eukaryotes as the amount of data increases.

UNITE uses CoL for overall eukaryotic taxonomy and classification. The taxonomic backbone of UNITE is flexible and allows web-based implementation of minor to major changes, such as those arising from publication of new or revised classification systems at any taxonomic level. For fungi, we use the Outline of Fungi (22) with some modifications (e.g. (23)). Expert users have similarly adjusted the classification in other groups of organisms—such as plants and oomycetes—to better reflect recent scientific results. New names and classifications are imported and verified as far as possible during the quarterly INSDC sequence import process using MycoBank (24) for fungi and some fungus-like groups, and CoL checklist, World Register of Marine Species (WoRMS; (25)) and Global Biodiversity Information Facility (GBIF; (26)) for the remaining groups of eukaryotes. In between these update sessions, users can add new names through a new import module available in PlutoF. This module fetches taxon names through a GBIF API (https://www.gbif.org/developer/summary).

### Database connectivity and data dissemination

UNITE has led the development of a third-party curation service in PlutoF to improve the value of public DNA sequences and their source metadata (e.g. material source, geolocation and habitat, taxonomic re-identifications, interacting taxa and literature). In collaboration with the European Nucleotide Archive (ENA; (27)), improved or corrected annotations of INSDC sequences residing in UNITE are fed back to primary repositories through the ELIXIR Contextual Data ClearingHouse (https://www.ebi.ac.uk/ena/clearinghouse/api) and shown on their record pages next to the original data (28,29). Searching and browsing of third-party annotations introduced by the UNITE Community can be done via PlutoF and ENA web services or by using the search interfaces of PlutoF and UNITE (Supplementary Figure S3). During 2023 alone, UNITE has contributed >4 000 annotations to ENA.

Metabarcoding is a major source of biodiversity data, and beginning in 2019, UNITE users have been able to publish metabarcoding datasets they manage in PlutoF through the Global Biodiversity Information Facility to become discoverable at the GBIF.org portal (https://www.gbif.org). These DNA-derived taxon occurrences are linked to UNITE SH identifiers that are incorporated in the backbone taxonomy of GBIF, meaning that also undescribed biodiversity is opened up for biodiversity data reuse and policy making along with biodiversity data from all other sources mediated through GBIF. Successive versions of UNITE SH classifications have been published and included in the GBIF backbone classification during the last few years (30), allowing users to compare and analyse datasets published with SH identifiers from different versions (versions 7–9) over time. To date, 10 datasets with >7M occurrence records linked to SH persistent identifiers have been published from PlutoF to GBIF. Re-annotations at the sequence level are also shared with the GBIF data portal, which facilitates the placement of SHs in the GBIF taxonomic backbone. This dual connection between the UNITE and the GBIF systems enables constant improvements of the quality of the sequence identification thanks to the evolving reference libraries.

## The UNITE website

Bioinformatics underpins much of UNITE, but we strive to make the data in UNITE easy to interpret, interact with and download also for non-bioinformaticians. While some expert tools and queries are reserved for users registered in PlutoF, a range of resources for sequence identification, query and analysis are openly available through the UNITE web portal. Our intention is to provide up-to-date, preformatted DNA sequence and metadata release files for any structured effort that needs these, and we offer such files for a number of tools, notably QIIME, mothur (31), BLAST (32), SINTAX and DADA2 (33). The underlying PlutoF sequence management platform offers registered users a comprehensive environment to manage biological collections, scientific studies and long-term datasets. All data and services of UNITE and PlutoF are provided free of charge.

The SH matching analysis (34) is a nascent digital service for global species discovery from environmental and other DNA sequence data. The tool places a user's unknown DNA sequences into existing UNITE species hypotheses or forms new SHs not yet present in the system, as applicable. Registered users can choose to imprint these (or some of these) new SHs into the SH system for public or personal use, according taxonomic permanence to what otherwise would have been very short-lived detections restricted to individual studies. The SH matching analysis output includes DOI-based identifiers and, if applicable, binomial names for communication of species hypotheses recovered from metabarcoding or Sanger data. The development version of the SH matching analysis is available as an EOSC-Nordic (https://www.eosc-nordic.eu) service for registered users, and the source code is available at GitHub (https://github.com/TU-NHM/sh_matching_pub).

## Outlook

A formidable challenge in eukaryotic microbiology is the immense number of dark taxa known exclusively from sequence data and defying any effort to isolate them. Current rules of nomenclature preclude formalization of these taxa (35,36), effectively curbing their inclusion in many biological contexts and pursuits. Integrating these taxa alongside formally recognized ones in a classification and naming system from the species to kingdom level and possibly beyond is needed to facilitate standardized and unambiguous communication. The UNITE taxon hypothesis system readily lends itself to this kind of representation, and we are currently exploring the use of artificial intelligence to produce a fully resolved pan-eukaryotic DOI-based taxon hypothesis release. Such a representation would ultimately allow plotting of metabarcoding datasets across the full eukaryotic tree of life. This, in turn, enables instant automatization of numerous challenging and hotly pursued research questions, for instance repeated detection of cross-kingdom co-occurrences of species to indicate previously overlooked ecological associations, or identification of the most similar communities from the pool of all available metabarcoding datasets.

In the near future the increasing read lengths of metabarcoding sequences will allow the full ribosomal operon rather than any of its individual components—the SSU (18S rRNA) and LSU (28S rRNA) genes and the intercalary ITS region—to be routinely targeted. While ribosomal sequencing has a long history in environmental microbiology, the available resources and repositories are essentially compartmentalized and tailored for each ribosomal component. Bridging these resources under a common naming system is highly desirable. This entails virtual assembly of full ribosomal sequences along with their metadata scattered across several separate databases—an undertaking that risks producing chimeric sequences and data. Assembled fungal genomes may offer guidance in this process, and UNITE recently assisted the EUKARYOME database (https://eukaryome.org) in the generation of a pan-eukaryotic, full-ribosomal chimera control reference dataset. We are exploring other avenues for merging data and metadata together with, e.g. the BOLD database (https://boldsystems.org). At present, we use ITSx to extract the ITS region from long-read metabarcoding sequences, after which the ITS component is incorporated into UNITE. Long-read metabarcode reads are thus used in UNITE, but their information content is not maximized.

By storing sequence occurrence data along with rich metadata on, e.g. locality and substrate of collection as well as interacting taxa, UNITE essentially offers a digital twin of eukaryotic biodiversity and communities worldwide. This virtual representation certainly presents technical challenges, but above all it encourages the life science community to rethink many current standpoints. It calls for a seamless two-way flow of information between metabarcoding and taxonomy, stresses the need for inclusion of as yet undescribed species and groups in all biodiversity-related efforts, and signals that the era when individual groups of organisms were routinely studied in isolation may well be over. Policies and protocols may not change overnight, but the looming biodiversity crisis forms a backdrop against which haste, for once, seems vital.

## Supplementary Material

gkad1039_Supplemental_File

## Data Availability

All sequences covered in this article are deposited in public sequence databases, INSDC and UNITE. Custom reference sequence datasets for a range of metabarcoding software pipelines are available for download on UNITE Resources page (https://unite.ut.ee/repository.php). UNITE SH datasets are published with DataCite DOIs and are accessible through UNITE (https://unite.ut.ee) and PlutoF (https://plutof.ut.ee) public homepages.
