# Supplementary material for: The UNITE database for molecular identification and taxonomic communication of fungi and other eukaryotes: sequences, taxa and classifications reconsidered
Source: Nucleic Acids Res. 2023 Nov 11;52(D1):D791–7. doi: 10.1093/nar/gkad1039 (PMC10767974; doi:10.1093/nar/gkad1039)
Supplement: gkad1039_Supplemental_File [file gkad1039_supplemental_file.pdf]

Supplementary Figure 1. Visualization of the *Rhizopogon verii* species hypothesis (SH) in UNITE 9.0.

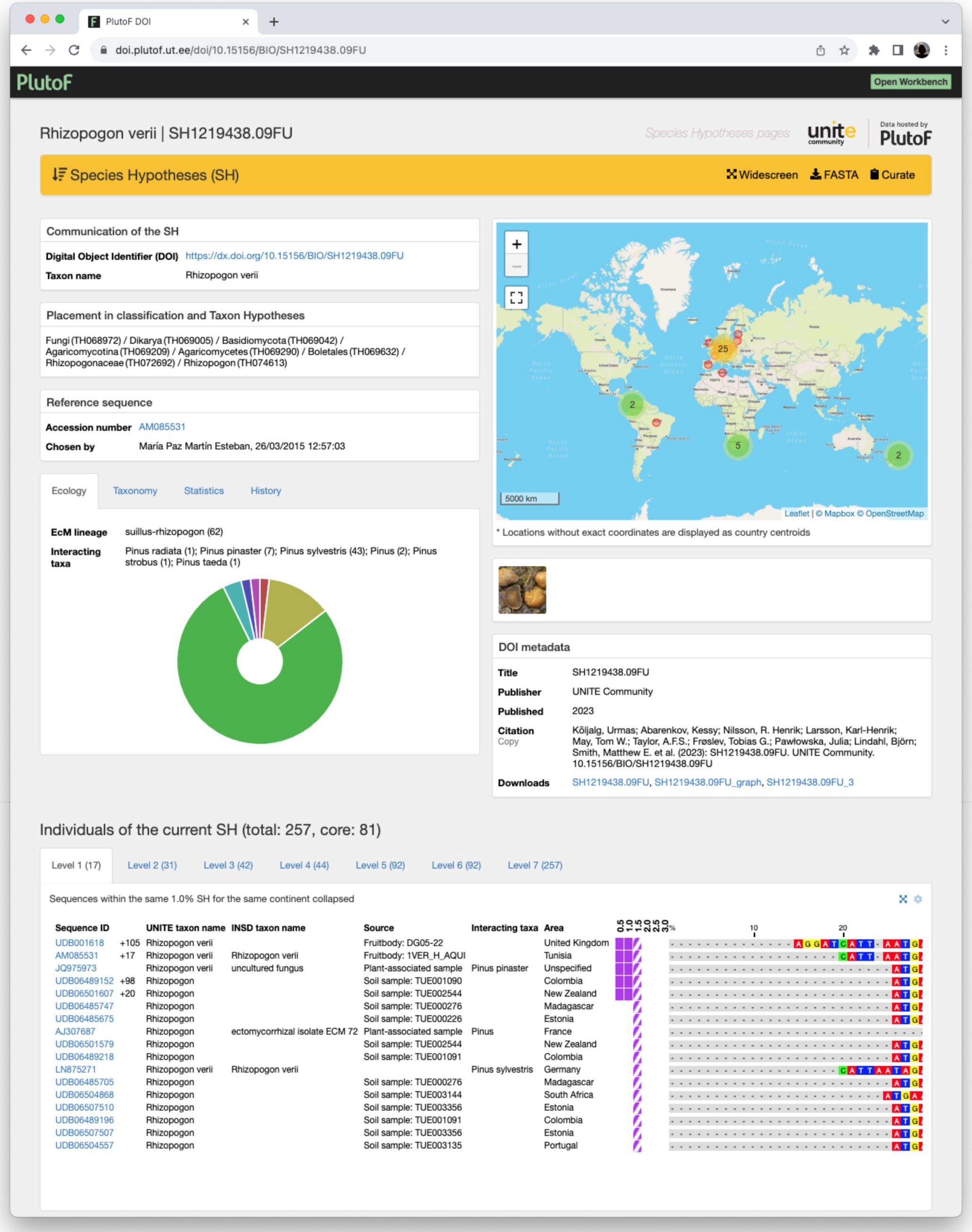

Supplementary Figure 2. Visualization of the taxon hypothesis (TH) of the fungal order *Boletales* in UNITE 9.0.

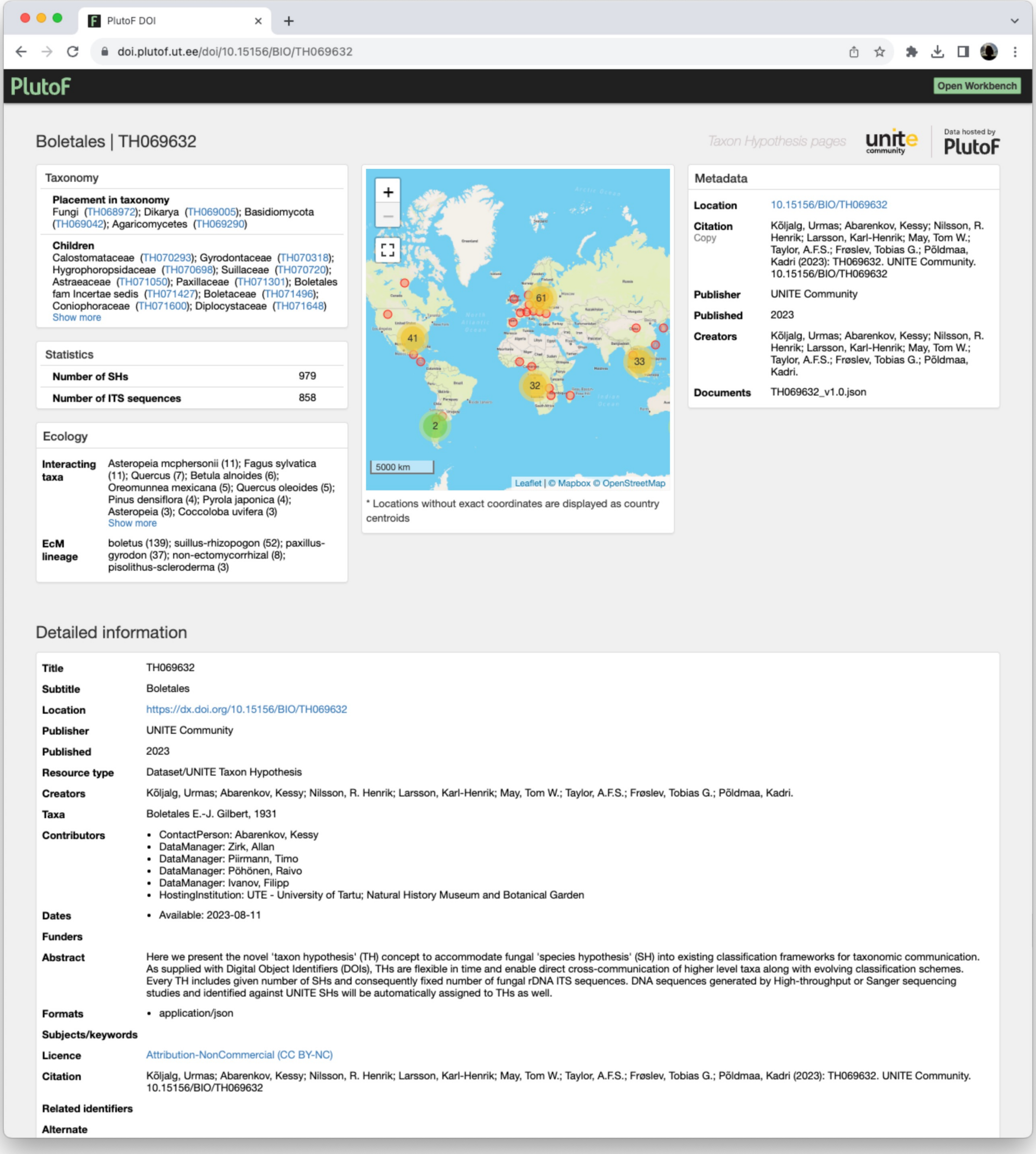
PlutoF DOI

doi.plutof.ut.ee/doi/10.15156/BIO/TH069632

PlutoF   Open Workbench

Boletales | TH069632   Taxon Hypothesis pages   unite community   Data hosted by PlutoF

Taxonomy

Placement in taxonomy
Fungi (TH068972); Dikarya (TH069005); Basidiomycota (TH069042); Agaricomycetes (TH069290)

Children
Calostomataceae (TH070293); Gyrodontaceae (TH070318); Hygrophoropsidaceae (TH070698); Suillaceae (TH070720); Astraeaceae (TH071050); Paxillaceae (TH071301); Boletales fam Incertae sedis (TH071427); Boletaceae (TH071496); Coniophoraceae (TH071600); Diplocystaceae (TH071648)
Show more

Statistics

**Number of SHs**	979
**Number of ITS sequences**	858

Ecology

**Interacting taxa**	Asteropeia mcphersonii (11); Fagus sylvatica (11); Quercus (7); Betula alnoides (6); Oreomunnea mexicana (5); Quercus oleoides (5); Pinus densiflora (4); Pyrola japonica (4); Asteropeia (3); Coccoloba uvifera (3) Show more
**EcM lineage**	boletus (139); suillus-rhizopogon (52); paxillus-gyrodon (37); non-ectomycorrhizal (8); pisolithus-scleroderma (3)

5000 km   Leaflet | © Mapbox © OpenStreetMap

* Locations without exact coordinates are displayed as country centroids

Metadata

**Location**	10.15156/BIO/TH069632
**Citation** Copy	Kõljalg, Urmas; Abarenkov, Kessy; Nilsson, R. Henrik; Larsson, Karl-Henrik; May, Tom W.; Taylor, A.F.S.; Frøslev, Tobias G.; Põldmaa, Kadri (2023): TH069632. UNITE Community. 10.15156/BIO/TH069632
**Publisher**	UNITE Community
**Published**	2023
**Creators**	Kõljalg, Urmas; Abarenkov, Kessy; Nilsson, R. Henrik; Larsson, Karl-Henrik; May, Tom W.; Taylor, A.F.S.; Frøslev, Tobias G.; Põldmaa, Kadri.
**Documents**	TH069632_v1.0.json

Detailed information

**Title**	TH069632
**Subtitle**	Boletales
**Location**	https://dx.doi.org/10.15156/BIO/TH069632
**Publisher**	UNITE Community
**Published**	2023
**Resource type**	Dataset/UNITE Taxon Hypothesis
**Creators**	Kõljalg, Urmas; Abarenkov, Kessy; Nilsson, R. Henrik; Larsson, Karl-Henrik; May, Tom W.; Taylor, A.F.S.; Frøslev, Tobias G.; Põldmaa, Kadri.
**Taxa**	Boletales E.-J. Gilbert, 1931
**Contributors**	• ContactPerson: Abarenkov, Kessy<br>• DataManager: Zirk, Allan<br>• DataManager: Piirmann, Timo<br>• DataManager: Põhönen, Raivo<br>• DataManager: Ivanov, Filipp<br>• HostingInstitution: UTE - University of Tartu; Natural History Museum and Botanical Garden
**Dates**	• Available: 2023-08-11
**Funders**	
**Abstract**	Here we present the novel 'taxon hypothesis' (TH) concept to accommodate fungal 'species hypothesis' (SH) into existing classification frameworks for taxonomic communication. As supplied with Digital Object Identifiers (DOIs), THs are flexible in time and enable direct cross-communication of higher level taxa along with evolving classification schemes. Every TH includes given number of SHs and consequently fixed number of fungal rDNA ITS sequences. DNA sequences generated by High-throughput or Sanger sequencing studies and identified against UNITE SHs will be automatically assigned to THs as well.
**Formats**	• application/json
**Subjects/keywords**	
**Licence**	Attribution-NonCommercial (CC BY-NC)
**Citation**	Kõljalg, Urmas; Abarenkov, Kessy; Nilsson, R. Henrik; Larsson, Karl-Henrik; May, Tom W.; Taylor, A.F.S.; Frøslev, Tobias G.; Põldmaa, Kadri (2023): TH069632. UNITE Community. 10.15156/BIO/TH069632
**Related identifiers**	
**Alternate**	

Supplementary Figure 3. Browsing and searching third-party curations added by the members of the UNITE community.

Example 1: Locality info and isolation source specified, taxon re-identification added for KJ180283.

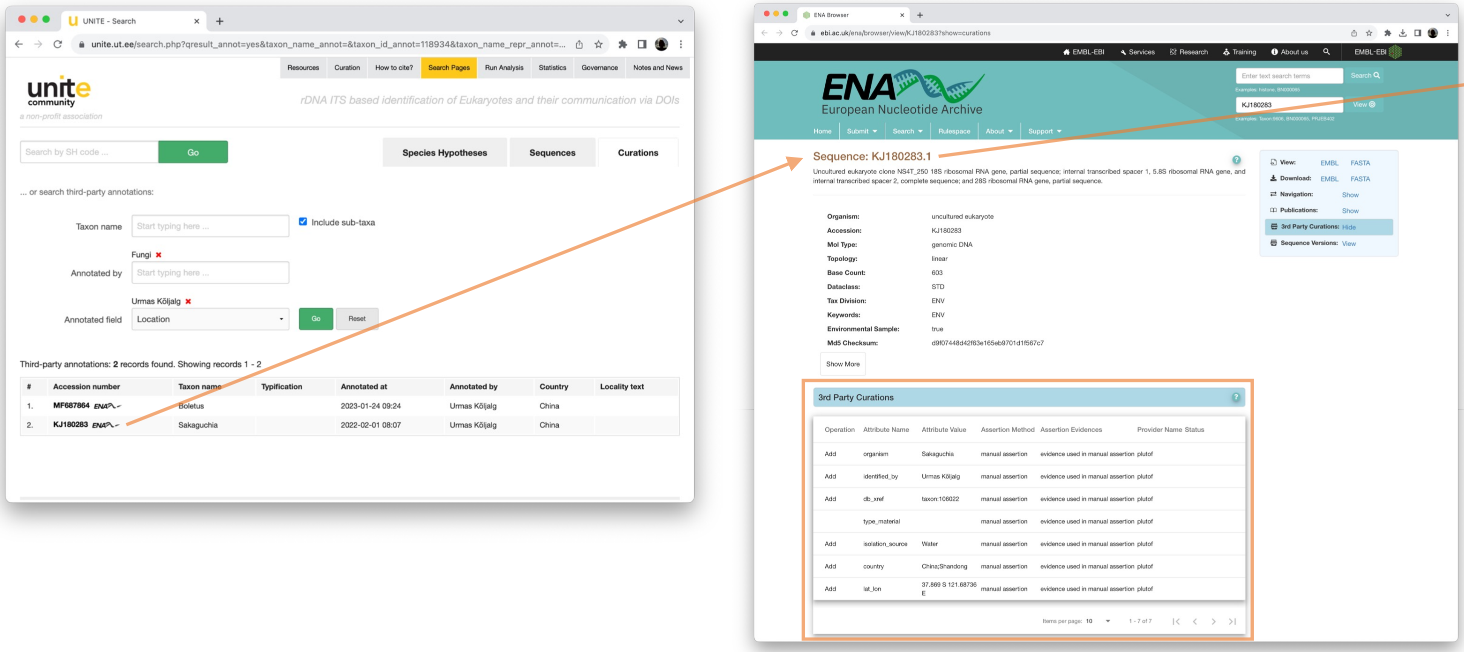

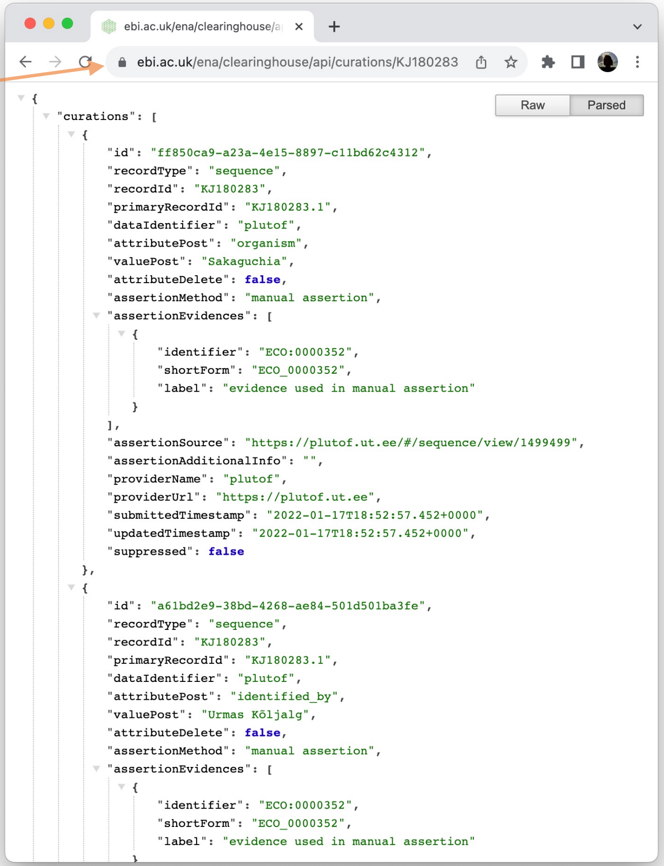

Example 2: Data on typification and newly described taxon name added for OQ073926.

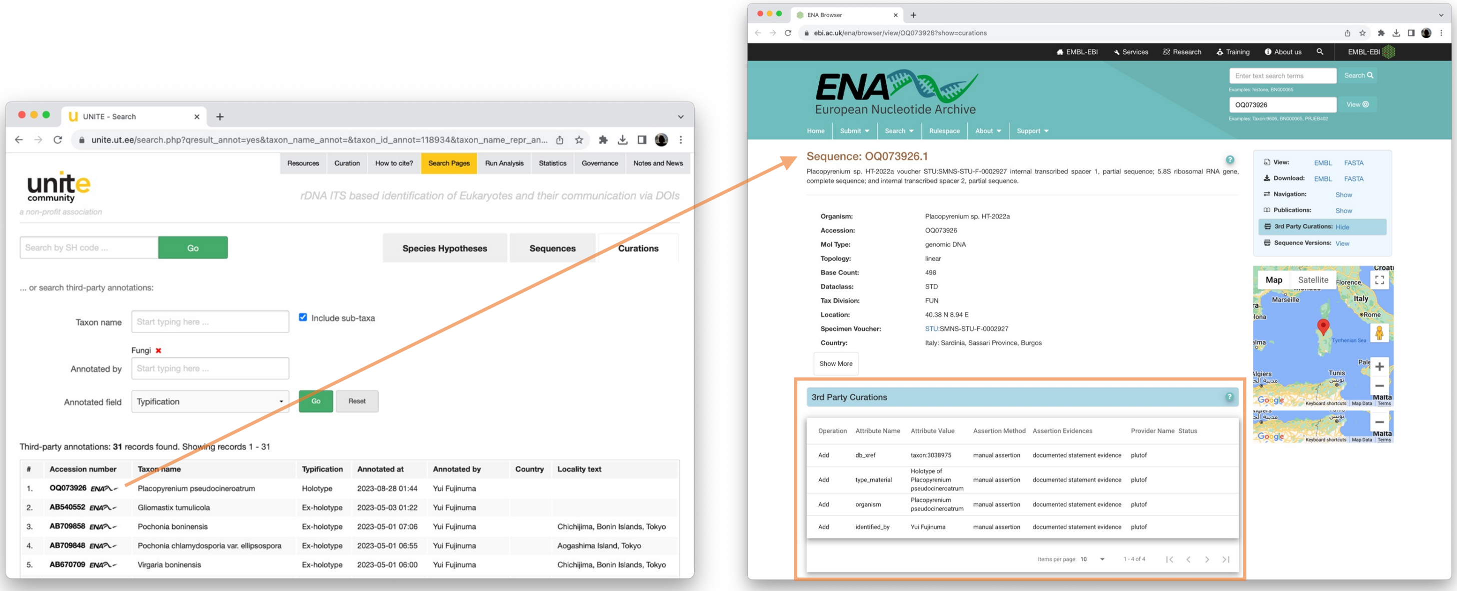

All third-party curations pushed to European Nucleotide Archive can be fetched programmatically using the Elixir Contextual Data ClearingHouse RESTful API - https://www.ebi.ac.uk/ena/clearinghouse/api/.
